# Motor-Cars as Ambulance Wagons

**Published:** 1914-10-31

**Authors:** 


### Motor-Cars as Ambulance Wagons.

THE ROYAL AUTOMOBILE CLUB SPECIFICATIONS.

There has been such an insistent call during the past weeks for additional motor ambulances for the Front that it is opportune to note the requirements adopted by the Royal Automobile Club for the conversion of the private motor-car for ambulance work.

These requirements were recently published in *Cooper's Vehicle Journal*—a trade journal—by whose kind permission we are able to quote some extracts:

"At the beginning of the war the idea was that each vehicle should be arranged to carry as many wounded as possible, but since then, in passing ambulances for service, the R.A.C. have strongly set their faces against the considerable rear overhang which was inevitable in some cases so long as bodies were applied indiscriminately to chassis. The body recommended is of the well pattern, and, with the floor-boards lifting up, is convertible into an extemporised waggonette—a feature of real importance, for it is found that the vast majority of wounded can be carried in this way. In the design the R.A.C. engineers have favoured a hoop-stick form of construction, which should be amply strong in view of the fact that the weight of the upper stretcher is partly taken by the centre pillars, and that the part of the load carried by the hoop-sticks only falls at a point not more than 2 ft. above the floor. As far as the stretcher accommodation is concerned . . . the possibilities of carrying not only the British Army pattern of stretcher, but those in use by other nationalities, have had to be considered.

"All methods of spring support or suspension have been discarded, consequently the lower stretchers rest on the floor, the upper on shelves or ledges."

The above extracts refer to a four-stretcher ambulance, but the R.A.C. have also prepared requirements for a two-stretcher body when the size of chassis will not permit of the larger ambulance being fitted. The "well" form of construction is employed in this also, although the "waggonette" form of accommodation is not provided. The space that naturally arises in the well on the stretcher side is utilised for locker accommodation.

